# A case report on ultrasound-guided pericardiocentesis with a right parasternal approach: a novel in-plane lateral-to-medial technique

**DOI:** 10.1186/s12245-024-00592-7

**Published:** 2024-02-01

**Authors:** Najem Abdullah Mohammed, Tanweer A. Al-zubairi, Moad H. Al-soumai

**Affiliations:** 1 Emergency Department and Intensive Care Unit, Al Zamalh Hospital, Mawia Street, Taiz City, Yemen; 2 Faculty of Medicine and Health Sciences, Taiz University, Habeel Street, Taiz, Yemen; 3 POCUS Academy, Sana’a City, Yemen

**Keywords:** Pericardiocentesis, Ultrasound-guided pericardiocentesis, High-frequency probe, In-plane technique, Lateral-to-medial approach, Right parasternal access, Point-of-care ultrasound

## Abstract

**Introduction:**

Emergency pericardiocentesis is a life-saving procedure that is performed to aspirate fluid from the pericardial space in patients who have severe pericardial effusion that is causing hemodynamic compromise. The current gold standard for pericardial fluid aspiration is ultrasound-guided pericardiocentesis. Echocardiography with a low-frequency transducer has generally been used in pericardiocentesis, but this method lacks real-time visualization of the needle trajectory, leading to complications. Therefore, we describe a case involving an ultrasound-guided pericardiocentesis method using a novel in-plane technique with a lateral-to-medial approach via the right parasternal and a high-frequency probe. The method was performed for an infant with cardiac tamponade.

**Case presentation:**

We present a case of a 14-month-old male infant who was brought to the emergency room with a history of cough, shortness of breath, and fever following recurrent chest infections. Despite prior treatments, his condition deteriorated, and signs of cardiac tamponade were evident upon examination. Cardiopulmonary point-of-care ultrasound confirmed the presence of a large pericardial effusion with tamponade. Emergency pericardiocentesis was performed using the novel in-plane technique, resulting in successful fluid aspiration and stabilization of the patient’s condition.

**Technique description:**

The proposed technique involves positioning a high-frequency ultrasound probe over the right parasternal area to obtain real-time visualization of the needle trajectory and surrounding structures, including the sternum, right internal thoracic vessels, pleural sliding end point, pericardial effusion, and myocardium. The needle is inserted laterally to medially at a 45-degree angle, ensuring safe passage between the pleural sliding endpoint and the right internal thoracic vessels while reaching the pericardial effusion.

**Conclusion:**

The presented technique provides real-time visualization of the needle and surrounding structures, which may potentially help to avoid complications and improve accuracy. The proposed technique may potentially enable access for emergency pericardiocentesis and for loculated pericardial effusion that has formed around the right atrium. Nevertheless, further studies with large patient populations are needed.

**Supplementary Information:**

The online version contains supplementary material available at 10.1186/s12245-024-00592-7.

## Background

Cardiac tamponade is a life-threatening condition that is characterized by the collection of fluid in the pericardial sac, which results in cardiac compression and impaired cardiac function. This condition can affect patients of all ages, but due to the smaller anatomical structures of pediatric patients, it poses unique challenges in such cases. Early diagnosis and treatment are essential for successful outcomes [1–3].

In cases of large, symptomatic pericardial effusion or cardiac tamponade, pericardiocentesis is the most useful therapeutic technique for early therapy or diagnosis [3, 4]. Emergency pericardiocentesis is a lifesaving procedure that is used to aspirate fluid from the pericardial space in patients with significant pericardial effusion that has resulted in hemodynamic compromise [5, 6]. The introduction of ultrasound-guided procedures has enhanced the technique’s safety and practicability and has contributed to its evolution over time [7, 8]. Ultrasound-guided pericardiocentesis has become the gold standard for the aspiration of pericardial fluid. First introduced in 1979 at the Mayo Clinic [7, 8], this method has been shown to be effective in comparison to blind or surgical methods [9].

Over time, this method has been refined into better techniques with different approaches, and with the increasing use of point-of-care cardiac ultrasonography, ultrasound-guided pericardiocentesis has become a viable option in the emergency department [10]. Several methods have been described, including parasternal, apical, and subxiphoid methods [7, 9–11], but the optimal approach for draining pericardial effusion remains controversial. One reason is that procedure selection frequently depends on the patient’s characteristics and the expertise of the hospital [9–25].

Conventionally, a low-frequency transducer has been used with echocardiography to diagnose pericardial effusion and determine the optimal puncture site [7–9, 11]. However, a few new nonconventional approaches to pericardiocentesis have been described, such as echocardiography-guided pericardiocentesis with a high-frequency transducer [12–15], an apical in-plane approach in a sitting position with a high-frequency transducer [12], and an in-plane medial-to-lateral technique through a left parasternal approach [13]. Real-time tracking of the needle using a high-frequency transducer enables clinicians to avoid injuring adjacent structures [12, 13, 16, 17]. The present study describes a new technique for pericardiocentesis using a high-frequency probe through a right parasternal approach. We also share our initial experience using this approach in the emergency room for infants with cardiac tamponade.

## Case presentation

A 14-month-old male infant presented with a history of coughs, shortness of breath, and fever 2 weeks prior. The symptoms were preceded by a recurring chest illness in the previous 2 months. He underwent treatment but did not improve. He was brought to the emergency room of our hospital due to breathlessness, irritability, fever, and poor feeding. His vital signs included a temperature of 37.9 °C, respiratory rate of 70 breaths per minute, heart rate of 171 beats per minute, blood pressure of 90/70 mmHg, and SPO2 of 89%.

**Fig. 1:**
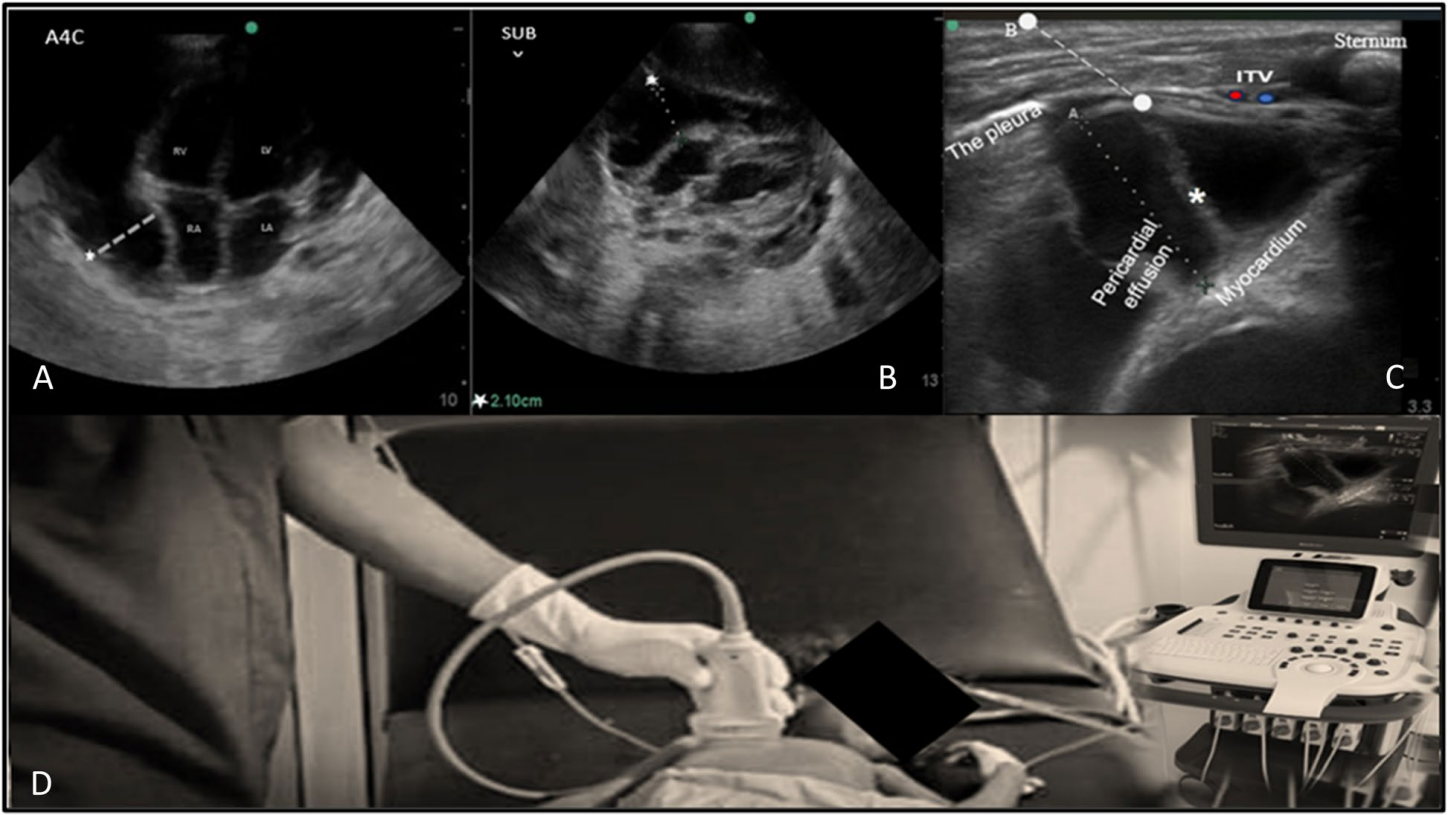
**A**, **B** Pre-procedural echocardiography views (apical 4-chamber and subxiphoid) revealing a large pericardial effusion concentrated maximally around the right atrium (dashed line and star). **C** Right parasternal ultrasonographic view displaying the pericardial effusion with fibrin strands, the surrounding vital structures (pleura, internal thoracic vessels (ITVs), and myocardium), the diameter of cardiac effusion *A*, and the path of needle insertion *B* (the distance from the skin to the pericardial space). **D** Image demonstrating the position of operator, ultrasound machine, and placement of high-frequency ultrasound probe in the right parasternal area

A physical examination revealed that the patient was ill and irritable. He had pallor, a low-grade temperature, dyspnea, tachycardia, congested neck veins, and a good peripheral pulse. A lung examination revealed small crackles in the left middle zone and limited air entry at the base. The results of a heart exam and other systemic exams were normal. Cardiopulmonary point-of-care ultrasound (POCUS) revealed significant pericardial effusion (Fig. 1) and left lung consolidation. A lab investigation revealed positive findings of a high white blood cell count (WBC) with lymphocytosis. His erythrocyte sedimentation rate (ESR) was 70 mm/h.

The patient’s condition worsened because of cardiac tamponade, necessitating an emergency pericardiocentesis. The procedure was performed using an in-plane technique with a lateral-to-medial approach via the right parasternal route with a high-frequency probe. After aspirating 60 ml of pericardial fluid, the patient’s hemodynamics stabilized, and he was monitored for 24 h with repeated cardiac scans (Fig. 3). A cytochemical test of the pericardial fluid revealed a high WBC, lymphocytosis, and a high protein level. The patient was then referred to a specific pediatric facility and diagnosed with tuberculosis. After 2 weeks, the patient was brought in for follow-up, and a cardiac scan indicated no pericardial effusion (Fig. 3). A repeat follow-up scan was done every month thereafter until the regimen of anti-tuberculosis drugs was completed, and the scans demonstrated no pericardial effusion.

## The technique

### Pre-procedure (finding the optimal puncture site)

**Fig. 2:**
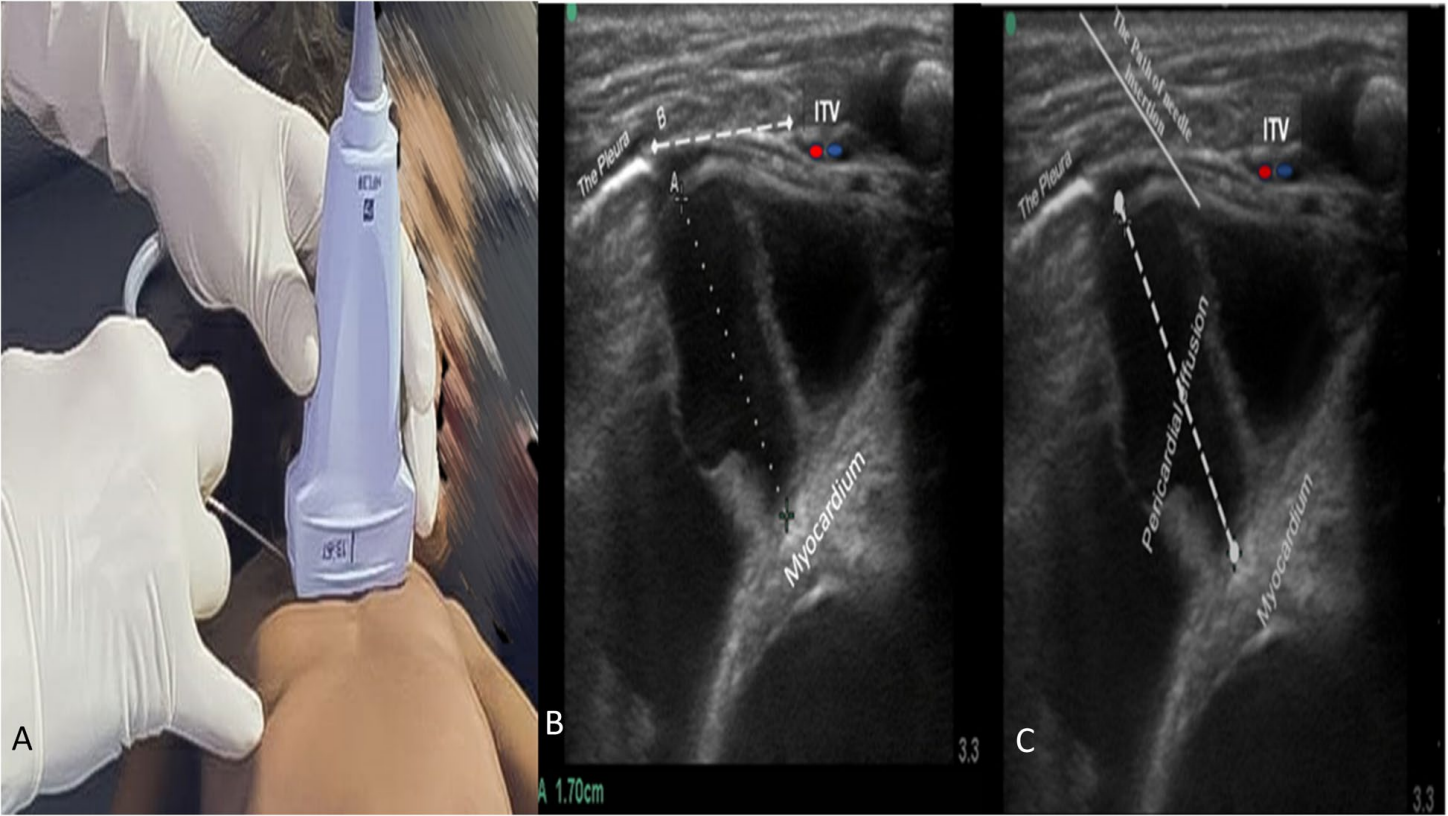
**A** Image demonstrating the operator handling the high-frequency ultrasound probe and needle insertion using in-plane technique with lateral-to-medial approach at a 45-degree angle. **B** Parasternal sonogram in long-axis view revealing the cardiac effusion diameter *A* and the distance *B* from the end point of the pleura sliding to the internal thoracic vessels (ITVs). **C** Parasternal sonographic long-axis view showing the needle’s path of insertion, which passes between the end point of the pleura laterally to the ITVs and medially to the pericardial sac and was visualized in real time during the procedure

The pericardial effusion was initially evaluated with a (cardiac) phased array probe for size and maximum collection location (Fig. 1A, B; Supplementary material files, video 1). The right parasternal view was then obtained with a high-frequency probe by putting it in a transversal position on the right parasternal area at the 4th intercostal space. The next step was finding and setting up the best view that showed the pericardium near the chest wall, through which the sternum, right internal thoracic vessels (ITVs), the end point of plural sliding, pericardial effusion, and the myocardium (right atrium) could all be easily seen. Measurements of the distance from the skin to the pericardium sac and effusion diameter were then taken (Fig. 1C; Supplementary files, video 2). The technique could be done safely with a pericardial effusion (PE) diameter and distance between the right ITV and plural sliding end points greater than 1 cm in the right parasternal window (Figs. 1C and 2A).

### Procedure

#### Patient and ultrasound positioning and preparation

All steps should be done under antiseptic conditions. An ultrasound machine was positioned on the left of the patient, who was kept supine throughout the procedure. The operator was on the right, allowing a direct view of the ultrasound screen after optimal adjustment of the ultrasound settings (Fig. 1C, D). Patients should be sedated to prevent unnecessary movements. The skin overlying the right chest was prepped, and the ultrasound transducer was covered with a sterile sheath.

#### Identifying the puncture site and surrounding structures

A high-frequency ultrasound probe was placed transversally in the intercostal space of the right parasternal (which was the area where the pre-procedure scan gave the best view). Then, the structures around it were identified, such as the sternum, right internal thoracic vessels, end point of plural sliding, pericardial effusion, and myocardium (right atrium). We then determined the optimal puncture path for drainage (Figs. 1C and 2A) (Supplementary material files, video 2). The optimal puncture path was defined as the pathway that had the lowest distance between the needle insertion site and the pericardial sac and avoided essential structures.

#### Needle insertion and confirmation

The operator fixes the liner probe on site with the left hand and the needle in the right hand and then smoothly advances the needle in-plane laterally to medially at a 45-degree angle. This is done with real-time visualization of the needle, which passes between the end point of plural sliding laterally and the right internal thoracic vessels medially to the pericardial effusion. The needle appears as a bright echogenic line in the long-axis view (Fig. 2C). Once the needle reaches the pericardial space, confirmation is obtained by aspiration of the pericardial cavity, as well as visualization of the tip of the needle in real time during the procedure. By inserting the needle along the superior border of the ribs, it is possible to avoid the intercostal vessels that run along the inferior border [18].

#### Fluid drainage

After confirming pericardial access, fluid drainage is initiated. This can be done by attaching a syringe or drainage system to the needle and gently withdrawing the fluid until hemodynamic stabilization is achieved. Throughout the procedure, continuous monitoring of the patient’s hemodynamic status is essential. An immediate improvement in hemodynamic stability is expected after successful pericardiocentesis.

### Post-procedure care

**Fig. 3:**
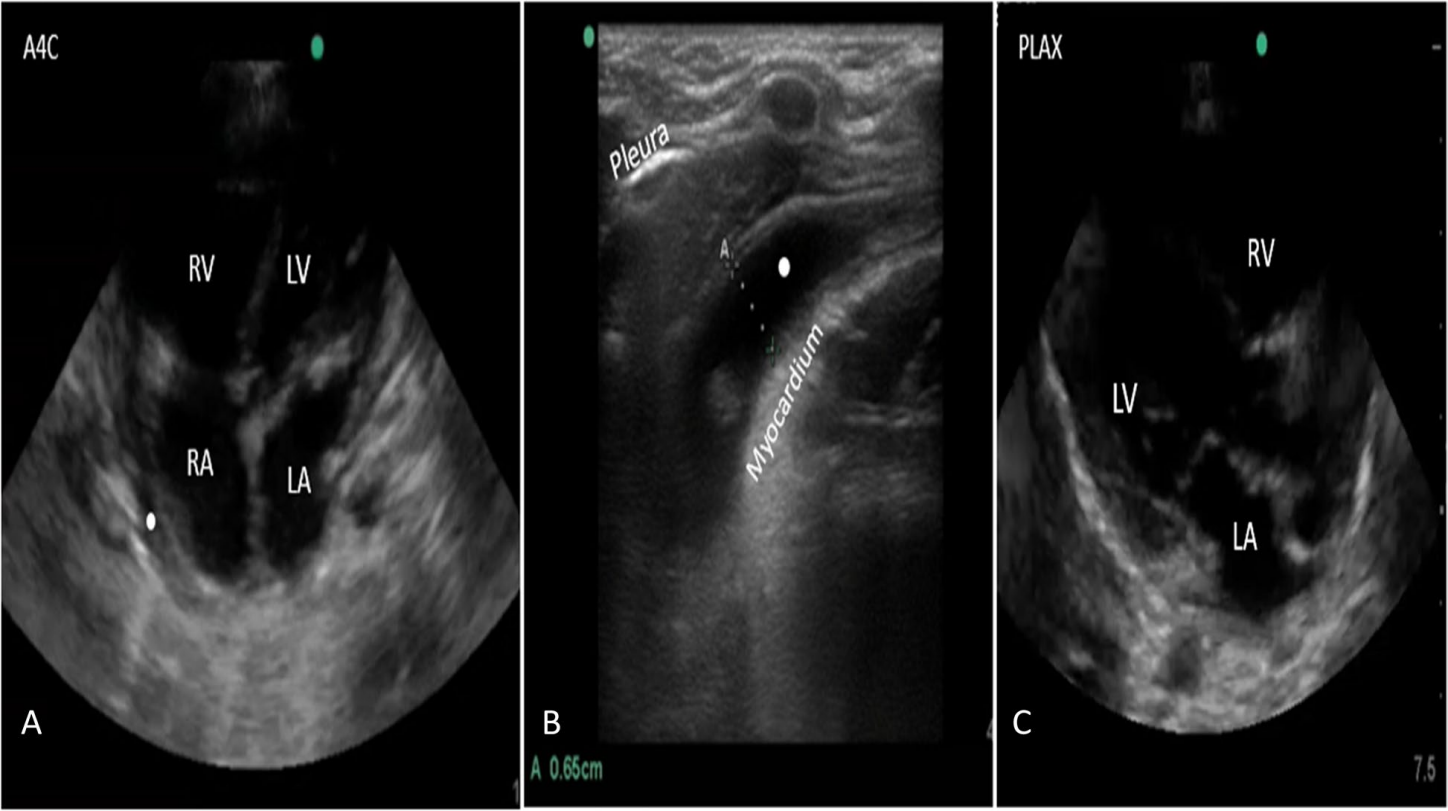
Cardiac ultrasonographic views following the procedure. **A** Apical four-chamber (A4C) view showing a decrease in the pericardial effusion (white dot). **B** Right parasternal view (longitudinal view axis) demonstrating a reduction in diameter of the cardiac effusion (white dot).The patient was referred to a specific pediatric facility and then diagnosed with tuberculosis. **C** Echocardiography scan and parasternal long-axis view revealing full clearance in pericardial effusion at 2 weeks after pericardiocentesis

After completing the pericardiocentesis, the patient is monitored for any complications or recurrence of pericardial effusion. Close observation and appropriate management are necessary to ensure a favorable outcome. This novel technique was used in an emergency situation with an infant with cardiac tamponade in an emergency room. It was performed at the bedside with the infant in a supine position and sedated, while vital signs and pulse oximetry were monitored. The pericardiocentesis was performed successfully without complications and relieved the hemodynamic instability (Fig. 3; Supplementary material files, videos 3 and 4).

## Discussion

This is the first report to describe technical experience of ultrasound-guided pericardiocentesis via the right parasternal route using a novel in-plane technique with a lateral-to-medial approach and a high-frequency ultrasound probe. The use of bedside ultrasound has significantly altered the practice of modern medicine. Greater familiarity with the technology has accelerated the transition of technical skill from novice to expert levels, enhanced performance in commonly encountered procedures, and led to novel approaches to clinical challenges [19–22].

In the 1970s, echocardiography-guided pericardiocentesis was developed and later accepted as the gold standard because it had fewer complications, such as liver, myocardium, artery, and lung perforation. Due to a lack of expertise in point-of-care ultrasound, many centers still use blind subxiphoid pericardiocentesis, which has a morbidity rate of about 20% and a mortality rate as high as 6% [7]. In contrast, 0.339% of echo-guided pericardiocentesis practitioners experience significant complications, while 0.420% experience minor complications [9].

The standard technique depends on identifying the location and distribution of pericardial fluid and inserting the needle at a site where the greatest amount of fluid is closest to the skin while using the “bubble test” to confirm the correct needle position. This technique is responsible for the low rate of minor and severe complications (3.5% and 1.2%, respectively) [9]. Alternative procedures with comparable complication rates, including the probe-mounted needle method, have been proposed with similar complications [23]. Pericardiocentesis guided by computed tomography (CT) is typically reserved for patients with poor acoustic windows and frequently complex, loculated pericardial effusions (typically posterior effusions), which can occur post-operatively and are not readily accessible via standard approaches. However, CT has limitations regarding accessibility and lengthy procedural durations, which were reported to be 65 min on average in one series [24].

In conventional techniques, low-frequency probes are typically used to route the needle insertion to areas with the greatest fluid accumulation and to ensure that the needle trajectory avoids vital structures [7–9, 11, 18, 26]. However, the complication rate is 5% [9]. Real-time visualization of the entire needle trajectory may be challenging with a low-frequency probe, which could result in injury to adjacent vital organs [13, 28]. Even with ultrasound guidance, several observational studies have demonstrated that the parasternal approach is preferable to the conventional subxiphoid approach as it offers the most direct, secure, and superficial access to the pericardial space [9, 15, 26, 27].

The apical or parasternal approach is transpleural and involves a risk of pneumothorax or infection spreading to the pleura and lungs. The subxiphoid approach increases the risk of liver, cardiac, and IVC injuries [11]. If echocardiography is available in an emergency situation, the intercostal approach can be used to perform urgent pericardiocentesis safely and effectively [28]. Conventional parasternal access is performed by inserting the needle directly next to the sternum to avoid damaging the ITVs that run about 1 cm laterally [23]. However, the technique involves a risk for potential complications due to a lack of visualization of the needle’s path and the adjacent anatomical structures.

To address the challenges of real-time visualization, a few new approaches using high-frequency probe US have been described [12–15]. High-frequency ultrasound is predominantly used in percutaneous thoracic interventions [16]. Using a linear probe enhances spatial resolution and reduces artifacts, allowing the operator to avoid injury to vital structures [29]. Case reports have described parasternal, in-plane, and real-time methods, but they did not describe them as lateral to medial via a right parasternal approach [14, 15]. Recently, Osman et al. [13] described using an in-plane medial-to-lateral approach via the left parasternal route for a small group of patients with a high-frequency probe, for which they reported a 100% success rate with no complications. The benefits of this medial-to-lateral technique include improved vision of the needle route and nearby anatomical structures, which can prevent complications and shorten the procedure duration.

The right parasternal access is beneficial for a number of theoretical reasons. First, the pericardial space around the right atrium is considered the typical site for the maximum collection of pericardial effusions because of gravity and having the lowest pressures of the cardiac chamber of the right atrium during the cardiac cycle. As a result, pericardial fluid accumulation is simpler in this location. Continually accumulating fluid causes the effusion to become circumferential.

Furthermore, because the right atrium has thin walls, it is the most susceptible to invagination due to the presence of a large pericardial effusion. This effect is most pronounced in the supine position, when effusion accumulates posteriorly around the right atrium [30–33]. As a result, the effusion diameter around the atrium will increase, and this site becomes the area of maximal fluid collection. Consequently, access to this collection around the right atrium via the right parasternal approach may potentially be easier and more feasible than with other approaches.

Second, loculated PE effusions and hematomas are the most common postoperative complications [24, 34]. Hematomas are typically found anteriorly and laterally of the right atrial free wall and could cause isolated compression of any chamber. This could result in hemodynamic collapse, especially if the affected chamber is adjacent to the atrium [34]. The right parasternal access has a direct path to the pericardial space around the right atrium, which suggests that the proposed technical approach may offer a feasible and valuable access point for loculated effusions or hematomas, which are inaccessible via standard approaches and require surgical intervention or CT-guided pericardiocentesis [24, 34].

Due to the parietal pericardium, a thin fibrous structure is closely adjacent to the lateral pleural surfaces [35]. With a symmetrically expanding pericardial contour around the heart, the pericardial layer comes into contact with the anterior thoracic wall in a large pericardial effusion, displacing the pleura laterally. In the present case, the right parasternal access was chosen because it was the clearest, closest, and safest with the greatest quantity of fluid collection (Fig. 1). Successful aspiration of pericardial fluid and stabilization of the patient’s hemodynamics were achieved (Fig. 3), suggesting potential benefits of the technique in similar situations.

The parasternal window effusion diameter should to be more than 1 cm to be suitable for the in-plane technique, and the thoracic vessels should to be mapped out with ultrasound before the procedure [18]. In the proposed approach, the needle advances between the right ITVs medially and the end point of plural sliding laterally. Thus, the distance between them should be sufficient for advancement and control of the needle on its way to the pericardial space while avoiding injury to surrounding structures, as well as complications. Our opinion is that more than 1 cm is a safe distance to perform the procedure (Fig. 2).

Many emergency physicians know how to use the in-plane technique for needle guidance with a linear array probe. This makes this less common procedure somewhat similar to more common procedures, such as ultrasound-guided peripheral and central vascular access [36]. Thus the described technique is potentially promising for emergency and critical-care physicians.

### The limitations and challenges

Operator experience and proficiency in ultrasound-guided procedures are essential for ensuring patient safety and favorable outcomes. Although many emergency clinicians use ultrasound-guided procedures on a regular basis, this is not the case for all of them. Another issue is that chest emphysema affects image quality. In addition, the parasternal approach is impractical in cases of cardiac arrest, and the subxiphoid approach is highly preferred [13, 23–28]. Lastly, only one clinical case was considered for this technical approach notation. Therefore, this novel approach requires validation with a larger population.

## Conclusions

This case report has demonstrated the clinical impact of a new technique, particularly in an emergency situation where alternative approaches may be limited or impractical. The use of real-time visualization potentially improves the accuracy of needle placement and reduces the risk of complications. This case report may serve as a basis for a new technical approach for pericardiocentesis, loculated pericardial effusion, or hematoma around the right atrium. Nevertheless, further research and clinical experience are required to validate the efficacy and prospective benefits with a larger patient population.

### Supplementary Information

**Additional file 1.**

**Additional file 2.**

**Additional file 3.**

**Additional file 4.**

## Data Availability

Not applicable.

## Abbreviations

POCUS: Point-of-care ultrasound
ITVs: Internal thoracic vessels
CT: Computed tomography
A4C: Apical four chambers
PLAX: Parasternal long-axis
SUB: Subxiphoid
